# Impact of Parenteral Maternal Supplementation with Trace Minerals and Vitamins on Neonatal Calf Antioxidant System and Growth in a Dairy Herd

**DOI:** 10.3390/ani14131868

**Published:** 2024-06-25

**Authors:** Evangelina Miqueo, Guillermo A. Mattioli, Dadin P. Moore, María G. Bilbao, Karen D. Moran, Alejandro E. Relling

**Affiliations:** 1 Departamento de Producción Animal, Facultad de Ciencias Agrarias, Universidad Nacional de Mar del Plata, Balcarce 7620, CP, Argentina; 2 Consejo Nacional de Investigaciones Científicas y Técnicas, Buenos Aires C1033AAJ, CP, Argentina; 3 Laboratorio de Nutrición Mineral, Facultad de Ciencias Veterinarias, Universidad Nacional de La Plata, La Plata 1900, CP, Argentina; 4 Facultad de Ciencias Veterinarias, Universidad Nacional de La Pampa, General Pico 6360, CP, Argentina; 5 Department of Animal Sciences, The Ohio State University, Wooster, OH 44691, USA

**Keywords:** fetal programming, dairy calves, oxidative stress, trace minerals, vitamin A, vitamin E, performance

## Abstract

**Simple Summary:**

Stress during the last third of gestation in dairy cows affects fetal development, which may result in reduction of birth weight and growth during the first month of life of the newborn calf. Mineral and vitamin supplementation can help to counteract the negative effects of stress and improve the development of the calf. This experiment aimed to assess the impact of injectable (subcutaneous) prepartum supplementation with copper, zinc, selenium, manganese, and vitamins A and E, on newborn calf growth and antioxidant capacity. Cows were assigned to one of two treatments groups: control (CG) or treatment (TG) and injected three times with saline solution or mineral and vitamins, respectively, before calving. Calves were monitored from birth to the ninth week. Feed intake, fecal score, body weight, average daily gain, body measurements, and parameters indicative of oxidative stress, were evaluated. Although calves in the TG ate more initially and had a lower fecal score than CG calves, there were no differences in body weight and no observed differences in their growth over the first two months. Parenteral supplementation of minerals and vitamins with antioxidant effects in prepartum dairy cows did not impact calf antioxidant system or growth in the first two months of life.

**Abstract:**

Oxidative stress may affect new born calves due to high stress suffered around birth. We hypothesized that maternal supplementation with micronutrients and vitamins in late gestation enhance the neonatal calf’s antioxidant system, decreasing the occurrence and duration of diarrhea, and improving growth from birth through weaning. To test this hypothesis, 80 multiparous cows were cluster-assigned to treatment groups. Treated group (TG) cows received mineral and vitamin supplementation while control group (CG) cows received saline solution. Feed intake and fecal score were measured daily until the ninth week. Weight and body measurements were registered weekly, and blood samples were collected from postpartum cows and calves after birth and at 7, 14, and 63 days of life. Although CG calves had greater fecal scores (*p* = 0.01), diarrhea characteristics did not differ. Calves in the TG showed greater starter intake (*p* = 0.04). Feed efficiency showed a trend with treatment-age interaction (*p* = 0.06). Calves in the CG had wider hips in the first week (*p* = 0.03), but not by the ninth week. Total antioxidant status, thiobarbituric acid reactive substances, and haptoglobin did not differ between treatment groups. Serum metabolites showed no differences. Supplementation did not impact calf antioxidant system or growth in the first two months.

## 1. Introduction

Over the course of decades, researchers have dedicated substantial efforts to unravel the intricate physiological transitions occurring in cows during the periparturient period [1,2,3]. Simultaneously, they have sought to devise effective management strategies aimed at mitigating stress during the crucial calving process [4,5,6]. It has been well documented that dairy cattle undergo several stressful periods throughout their life cycle, which can lead to an increase in metabolism and the production of excessive free radicals [7,8,9,10]. Excessive free radicals can cause damage to cell membranes, DNA, and other critical cell and tissue components [11]. As a result, the immune system may weaken, affecting the overall health, growth, development, reproduction, and milk production of the animals [12,13,14].

Numerous experiments have highlighted the adverse effects of oxidative stress on cattle, particularly during the transition period and early postpartum stages [15,16,17]. However, it is increasingly recognized that various situations that affect the cow during late gestation have enduring effects, influencing aspects of calf metabolism at birth and immunity post-colostrum intake [18]. Furthermore, oxidative stress during the transition period impacts the developing fetus [14,16,19]. This can alter the offspring’s ability to defend themselves against pathogens and cope with stressors, resulting in changes in body weight during the critical first month of life [14,19,20]. In this sense, fetal programming is a crucial consideration in dairy cattle since maternal oxidative stress not only affects immediate postpartum health but also has long-term implications for calf development and resilience [21]. Understanding and mitigating oxidative stress during pregnancy is vital for optimizing dairy cattle health and productivity.

Additionally, newborn calves must adapt to the challenges of extrauterine life, experiencing physiological and environmental changes that can make them vulnerable to the negative effects of oxidative stress, affecting their vitality [8,20,22]. In addition, newborn calves, grappling with an underdeveloped immune system, rely immunologically on the successful passive transfer of maternal immunoglobulins (Ig) from colostrum post-birth [23,24,25].

Trace minerals such as selenium, copper, zinc, and manganese play crucial roles in the antioxidant defense system as co-factors for certain enzymes, while vitamin E acts directly as an antioxidant, protecting plasmatic membranes from oxidative damage, and vitamin A acts indirectly by stimulating gene transcription of antioxidant mechanisms [26,27,28]. Maternal supplementation with organic trace minerals has been shown in previous experiments to positively impact the calf’s immune system, metabolism, antioxidant profile, and growth [29]. While certain studies have explored oral mineral supplementation for dairy cows during the prepartum period and its effects on immunological parameters and calf body weight [25,29,30], beef cattle have garnered more attention in this area [31,32]. Some researchers have noted a decrease in mineral absorption during stressful periods like the transition period for dairy cows [33]. Consequently, there exists a notable research gap concerning the impact of mineral and vitamin supplementation on Holstein cows, on the antioxidant capacity of calves in the first two months of life, and how this affects the occurrence of diarrhea, calves’ birth weight, and daily live weight gain.

The overall hypothesis of this experiment is that maternal supplementation with trace minerals and vitamins associated with the antioxidant system, administered parenterally in the dry-off period enhances the neonatal calf’s antioxidant system, and reduces the products of lipid peroxidation as a result of a reduced oxidative status. This enhancement in the antioxidant system is reflected in the occurrence of fewer cases of diarrhea, with later onset of this metabolic disturbance, lower average fecal score and duration of diarrhea, as well as greater rates of daily weight gain and growth measures from birth through weaning. In this experiment, we aimed to evaluate whether parenteral maternal supplementation with trace minerals and vitamins during late gestation impacts neonatal calf’s antioxidant capacity, daily weight gain, and growth measures during the first two months of life in a dairy herd.

## 2. Materials and Methods

The experimental procedures were conducted in accordance with the protocol approved by the Institutional Committee for the Care and Use of Experimental Animals at the National Institute of Agricultural Technology (INTA CeRBAS), Argentina (Permit No. 226/2021).

### 2.1. Experimental Design and Maternal Treatments

The study was conducted from December 2020 to July 2021 in a commercial dairy farm located 25 km from Tandil, Buenos Aires, Argentina (37′15671227638485° S, 59′11661255216904° W). A total of 80 pregnant multiparous Holstein cows with parity number ranging between 2 and 5, were included in the study and blocked according to drying-off characteristics. Animals were allocated to two treatment groups through a cluster analysis, with the objective to achieve maximum homogeneity in each treatment group. Once the multivariate proximity of each animal with its closest neighbors was established using the “average linkage” method within the sample, the treatment (TG) was applied randomly to one of the cows, leaving its nearest neighbor as a control (CG), and so on, until completing 40 treated animals and 40 control animals. In this way, the local control of the experimental units was maximized, trying to balance both treatment groups in relation to their physiological-productive characteristics.

The treatment groups were treatment where cows were parentally infused with minerals and vitamins that have important roles in the antioxidant defense system (TG, *n* = 40), or control (CG, *n* = 40), where cows were parenterally infused with saline solution. Cows in both treatment groups were maintained under identical conditions throughout the study.

On the dry-off day (60 days before the expected calving date), as well as 30 and 9 days prior to the expected calving date, cows in the TG received parenteral supplementation of minerals and vitamins (5 mL of each, subcutaneously), while the CG received the same volume of sterile saline solution. The mineral supplement contained Cu (10 mg/mL as edetate), Zn (40 mg/mL as edetate), Se (5 mg/mL as sodium selenite) and Mn (10 mg/mL as edetate), and the vitamin supplement contained 3.5% vitamin A retinyl palmitate and 5.0% vitamin E acetate (Adaptador Min^®^ and Adaptador Vit^®^, respectively, Biogénesis Bagó SA, Garín, Argentina).

During the dry-off period, all cows were provided with the same diet. Up until 30 days before calving, their diet consisted primarily of fescue and white clover pasture. However, starting from 30 days prior to calving, the cows were relocated to the pre-calving pen, where they were given 14 kg (dry matter basis) of a total mixed ration, which included whole-plant corn silage, along with wheat straw hay, and anionic salts. During the entire time, cows had unrestricted access to clean water.

### 2.2. Management of Cows and Calves

As cows began showing imminent signs of calving (e.g., restlessness, isolation from the herd, visible udder enlargement, edematous and dilated vulva, or appearance of a clear discharge from the vulva), they were carefully transferred to a maternity pen (50 m × 100 m). Following parturition, the cows were moved to a milking parlor where they were milked within the first hour after giving birth. Colostrum immunoglobulin content was assessed using a Brix Refractometer (Atago PAL-1 Refractometer).

After birth, calves were weighed, their navels were disinfected with a 7% tincture of iodine solution and separated from the dam. Calf sex was registered. Within the first 4 h of life, calves were provided with 3.8 L of colostrum. Calves were bottle fed with colostrum from the dam when it registered a measurement equal to or greater than 24° Brix on the digital refractometer. Alternatively, if the maternal colostrum did not meet this criterion or in the presence of mastitis or other sanitary parameters, calves received colostrum from a colostrum bank. The selection criteria for the banked colostrum included meeting specific quality standards, such as measuring total proteins on the Brix refractometer or adhering to stringent sanitary parameters, including the absence of mastitis. All calves involved in the trial consumed all of the colostrum supplied. Following the first meal with good quality colostrum, calves were provided with a subsequent meal comprising similarly excellent colostrum. This was followed by two additional meals each offering colostrum of intermediate quality. Once colostrated, calves were individually housed and offered unlimited access to both water and a starter concentrate (Ruter^®^, ACA, Buenos Aires, Argentina) for the first 21 days. Following this period, they transitioned to another commercial concentrate (Mamon, Cooperativa Agorpecuaria de Tandil Ltd.a., Buenos Aires, Argentina) until the end of the trial (Table 1).

Calves were fed 6 L of whole milk daily, divided into two meals, until day 56 when weaning occurred. They remained on the stake for an additional week to monitor their consumption and weight gain during that period. Daily records were maintained for milk and concentrate intake.

Health checks were recorded once a day, along with rectal temperature, respiratory rate (movements per minute, mpm), and heartbeat (bpm), always at the same hour until the end of the trial, according to the scale proposed by the University of Wisconsin [34]. Fecal scores were assessed by a single observer using a scale from 1 to 5 according to the fluidity, every day before the morning feeding [35]. When fecal score was equal or greater than 3 it was considered that the calf had diarrhea, and an oral electrolyte solution was offered 2 h after milk feeding until the fecal score was ≤2. Calves that presented fever, apathy, or loss of appetite, received antibiotic therapy according to the recommendation of the responsible veterinarian. Additionally, cases of pneumonia were documented, including their duration and corresponding treatment.

### 2.3. Measurements and Sample Collection

All calves were evaluated 48 h after birth for transfer of passive immunity using the Atago PAL-1 digital Brix refractometer. For this determination, using tubes containing clot activator, a blood sample was taken from each calf from the jugular vein 48 h after birth. After centrifugation, serum was separated, and serum protein concentration was determined indirectly using the above-mentioned Brix refractometer. Passive immunity transfer was considered successful when the digital refractometer reading was greater than or equal to 9.4° Brix [36].

Feed samples were collected from the prepartum pasture, and from the total mixed ratio that prepartum cows received during the last month of pregnancy. Also, samples from the starter concentrate fed to calves were collected during the experimental period and frozen at −20 °C for chemical composition analysis (Table 1). Nutritional profile was determined by the near-infrared spectroscopy method in a commercial laboratory (Rock River Laboratory Inc., Santa Fe, Argentina). Briefly, feed samples were dried and ground to determine dry matter (DM) percentage [37]. The sample was placed into a cup with a glass bottom and a reflectance instrument was used to bounce near-infrared (NIR) light off the sample. The reflected light was measured, creating a graph, with wavelength on the x-axis and absorbance on the y-axis. The nutrient and digestion values for the sample were measured (predicted). The absorbance measures were related to nutrient and digestion properties for the sample. The NIR calibrations were supported with wet chemistry.

Before the morning feeding, weekly from birth to the ninth week of life, calves were weighed using a mechanical scale and growth measurements, such as withers height and hip width, were also taken using a ruler with a scale in centimeters, and heart girth using a bovine metric tape, with a scale also in centimeters. Blood samples were collected by puncture of the coccygeal vein in the cow before calving and immediately after calving, and from the jugular vein of the neonate immediately after birth and at 7, 14, and 63 days of life, always two hours after morning feeding. Blood samples were stored in tubes with a clot activator and kept at room temperature until centrifugation (~30 min) to obtain serum. Subsequently, serum samples were aliquoted and stored at −20 °C until further analysis of glucose, total protein, albumin concentration, total antioxidant status (TAS), thiobarbituric acid reactive substances (TBARS), and haptoglobin concentration.

### 2.4. Blood Metabolites and Oxidative Stress Biomarkers

Serum samples from calves immediately after birth and at 7, 14, and 63 days of life were enzymatically analyzed for the determination of serum glucose, total protein, and albumin by using a Wiener Autoanalyzer CM 250 and commercially available kits following the manufacturer’s instructions (Wiener Laboratories, Rosario, Argentina). 

To evaluate oxidative stress, TAS and TBARS (Antioxidant Assay Kit and Assay Kit, respectively, Cayman Chemical Company, Ann Arbor, MI, USA) were used. These assays were previously validated for bovine [38]. Both variables were measured in 15 randomly selected pairs of cows and their calves per treatment group (15 cows and their calves from TG and 15 cows and their calves from CG, corresponding to the same pair group). Serum samples from postpartum cows and calves immediately after birth and at days 14 and 63 of life were analyzed using commercial kits. Samples and reagents were prepared according to the manufacturer’s recommendations. Serum samples from the same animals were also analyzed to determine haptoglobin concentration, using a haptoglobin-turbitest assay (Wiener Laboratories, Rosario, Argentina). The basis of the method is that the haptoglobin present in the sample binds to the specific antibody provided in the reagent, forming insoluble immune complexes that cause turbidity, quantifiable at 340 nm. Turbidity is directly proportional to the presence of haptoglobin in the samples. The determinations were conducted on the Intelligent Clinical Chemistry Analyzer (InCCA, DICONEX S.A., Quilmes, Buenos Aires, Argentina). To calibrate the equipment, a four-point curve was generated from the High–Level Protein Calibrator (Wiener Laboratories) and physiological solution NaCl 0.9% m/v. Quality control was carried out using the High-Level Protein Calibrator (Wiener Laboratories) undiluted, according to the manufacturer’s recommendations [39].

### 2.5. Statistical Analysis

A randomized complete block design was employed with 80 multiparous Holstein cows based on parity number, previous lactation milk yield, body condition score, and expected day of parturition. To achieve maximum homogeneity within treatment groups, cluster analysis was performed on these variables using the average linkage method. This statistical technique establishes the multivariate proximity between cows, enabling precise pairing for treatment assignment. The treatment group with supplementation was assigned to a specific cow, while their closest neighbors were designated to the untreated control group, continuing this pattern until 40 treated and 40 untreated animals were assigned. This method aimed to maximize local control of the experimental units, balancing both groups in terms of physiological and productive characteristics. Potential confounding variables, such as environmental conditions and genetic factors, were controlled by incorporating the expected day of parturition into the randomization process, ensuring that cows calving at similar times were equally distributed between treatment and control groups. All analyses were performed using R statistical software, version R-4.3.1 [40].

The number of animals was selected based on power analysis of the results of previous work on fetal programming. Different numbers of animals were selected for the different determinations. For each variable, we considered the variability of the response variable, a power of 80% and an alpha value of 0.05. For offspring body weight, skeletal growth, intake, feed efficiency, fecal score, and diarrhea characteristics (n = 40), TBARS (n = 15), TAS (n = 15), haptoglobin (n = 15), glucose (n = 15), total protein (n = 15), and albumin (n = 15), were deemed adequate [38,41].

For repeated measures as body weight, average daily gain, body growth measures, milk, starter, total intake, feed efficiency, fecal score, and blood parameters, a linear mixed-effects model was implemented by using the lme4 package in R, to analyze the trajectories of calves under different experimental conditions over time. The model incorporated fixed effects to evaluate the influence of the treatment, time (in weeks), and sex on the response variable, including a quadratic term (when applicable) to capture potential non-linear growth patterns, and the interaction treatment by age. The sire was included in the model as a covariate whenever applicable during the analysis of variables related to calves, recognizing the potential genetic influence on the response variables. Random effects were included to capture individual variation among the calves and to accommodate the autocorrelation of repeated measures within the same calf. Explanatory variables were retained in the final model if they achieved statistical significance at a likelihood ratio test *p* value of 0.05 or less. Overall model fit was based on the Akaike Information Criterion (AIC), Bayesian Information Criterion (BIC), and visual assessment of Pearson’s residuals against fitted values, Q-Q standardized residuals against standardized normal quantiles, to ensure assumptions of normality were not violated.

For data without repeated measures, the model included the effect of treatments (T) and sex as fixed effects, and the sire was included as a covariate whenever applicable during the analysis of variables related to calves. This approach ensures that the analysis accounts for potential confounding factors and provides a robust evaluation of the treatment effects.

A survival analysis technique was used to estimate the days until the first episode of diarrhea. The Cox proportional hazards regression model was adjusted for time (days to diarrhea, fecal score greater than or equal to 3). A Kaplan–Meier survival function was used to estimate the time to onset of diarrhea based on the treatment received by the mother (TG or GC). The median time to onset of diarrhea between the two treatment groups was compared using a log-rank test to test whether they were affected by the treatment received by the mother (TG or CG; at *p* ≤ 0.05).

The data were presented as least squares means and accompanied by a standard error of the mean. Finally, significant differences were declared at *p* ≤ 0.05, and a tendency for significance was set at *p* ≤ 0.10 and >0.05.

## 3. Results

### 3.1. Descriptive Data

Out of the 80 cows enrolled in the study, a total of 84 calves were born (45 females and 39 males), with four cases of dystocia at birth recorded, two from each treatment group. Four cows, two from the CG and two from the TG, gave birth to multiple calves, but these specific animals were excluded from blood analysis. Instead, we focused solely on recording their growth, weight gain, health parameters, and consumption patterns. In addition, two calves, one from the CG and one from the TG, died. The calf from the CG died from unknown causes during its first week of life, and the calf from the TG died due to pneumonia during its eighth week of life. The data pertaining to those two calves were not included in the subsequent analysis, just for body weight at birth.

All calves received the first feed of good quality colostrum within the first 3 h of life, and all of them were successful in the transfer of passive immunity, measured with a Brix refractometer.

### 3.2. Fecal Score, Onset, Duration, and Severity of Diarrhea

**Figure 1:**
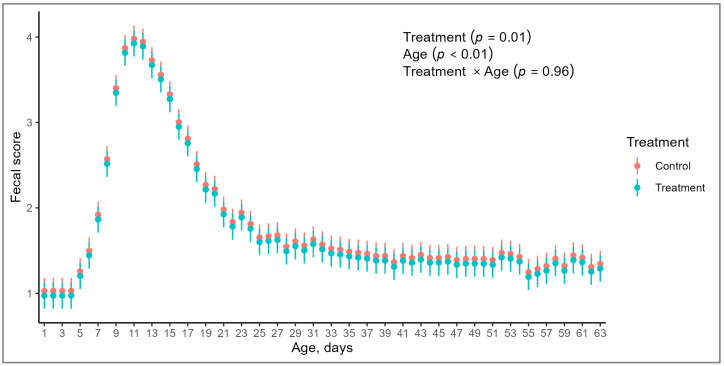
Fecal score in Holstein calves, from birth to 63 days of life, born from cows with (TG) or without (CG) supplementation with mineral and vitamins during the prepartum period.

The fecal score was not affected by the interaction of treatment and age of calves (*p* = 0.96; Table 2). However, there was an effect of age on the fecal score (*p* < 0.01), with greater scores during weeks two and three of age, commonly defined as the period of greater occurrence of diarrhea (Figure 1). Additionally, calves in CG exhibited greater fecal scores than their counterparts in the TG (*p* = 0.01; Table 2). No treatment differences (*p* ≥ 0.1) were noted in the onset of diarrhea, duration of the episodes, number of diarrhea episodes, and the intensity of diarrhea (Table 2), defined as the number of days with a fecal score greater than three.

There was no interaction between treatment and age of the animals, and also no differences were observed on rectal temperatures between treatments (*p* = 0.38; Table 2), and this parameter decreased throughout the trial (*p* < 0.01; Table 2). The maximum rectal temperature was observed during the first and second week of life, accompanying the greatest fecal scores observed in the same period. Respiratory rate did not differ between treatments and ranged from 44.8 mpm during the first week of life, noting a reduction to 30.7 mpm to the ninth week of life (*p* < 0.01; Table 2). Heart rate also did not present differences between treatments, but there was an age effect, with the maximum values of heart rate (125 bpm) observed in the first week, with a gradual decrease up to the end of the milk rearing, with 110 bpm (*p* < 0.01; Table 2).

### 3.3. Milk, Calf Starter, and Total Intake

**Figure 2:**
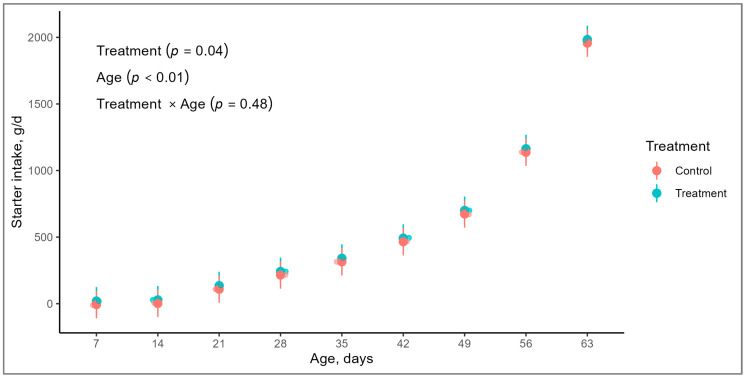
Evolution of starter intake (g/d) of Holstein calves, from birth to 63 days of life, born from cows with or without supplementation with mineral and vitamins during the prepartum period.

No differences were observed for milk intake between treatments (*p* = 0.14; Table 3). As expected, milk intake was different throughout the experimental period, since in the ninth week calves stopped receiving milk (*p* < 0.01). Starter intake showed no treatment by age interaction (*p* = 0.48), but calves from the TG presented greater starter intake, compared with calves in the CG (*p* = 0.04; Table 3). Furthermore, there was an age effect on starter intake, which increased from the first week of life onward (*p* < 0.01; Figure 2). Regarding total dry matter intake (solids from whole milk and starter), there was observed a tendency in total dry matter intake between treatments, in favor of calves in the TG (*p* = 0.06), and there was an increase from the first week of life onwards, in total dry matter intake (*p* < 0.01).

### 3.4. Body Weight, Average Daily Gain (ADG), Feed Efficiency, and Growth Measures

**Figure 3:**
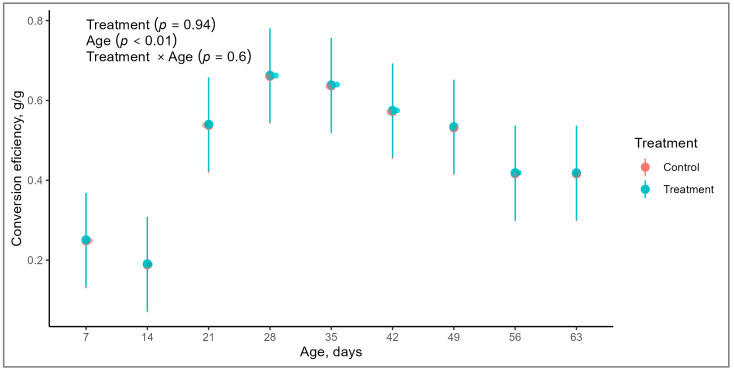
Evolution of feed efficiency (ADG/total DMI) of Holstein calves, from birth to 63 days of life, born from cows with or without supplementation with mineral and vitamins during the prepartum period.

There was a tendency in calves’ birth weight due to treatments (*p* = 0.08; Table 3), but we observed no treatment differences (*p* = 0.57) in body weight at the ninth week of life. Moreover, no differences were observed in ADG between calves born from cows in TG and those born from CG cows (*p* = 0.76). Notably, ADG increased as the experimental period advanced, with the most substantial daily gains occurring during the last three weeks prior to weaning (*p* < 0.01). Feed efficiency, calculated as the ratio between average daily gain (ADG) and total DMI, exhibited a trend to the interaction between treatment and age of calves (*p* = 0.06). Differences in feed efficiency due to the age of the animals were observed (*p* < 0.01; Figure 3).

In terms of skeletal measurements, there were no interactions between treatments by age of the calves for hip width, wither height, and heart girth, but calves in CG exhibited wider hips (*p* = 0.03). Also, there was an age effect for hip width, progressively increasing throughout the trial (*p* < 0.01). Wither height and heart girth did not differ (*p* = 0.94 and *p* = 0.11, respectively) between treatments. But an age effect was observed, as expected (*p* < 0.01; Table 3). There were also no differences in the increase in growth measures throughout the experimental period, as observed in Table 3.

### 3.5. Serum Metabolites, Antioxidant Capacity, and Lipid Peroxidation Biomarkers in Cows and Calves

There were no observed differences between TG and CG, on serum TAS, TBARS, and haptoglobin, on the cows immediately postpartum (*p* ≥ 0.05; Table 4).

When observing these oxidative stress biomarkers on calves, we observed no interaction between treatments and age at the sampling, for TAS, TBARS, and haptoglobin (*p* ≥ 0.05). When observing TAS concentration at birth and at the ninth week of life, no differences between treatments were found (*p* = 0.11, *p* = 0.13, respectively). But there was an age effect, increasing the TAS concentration towards the last sampling, and the lowest concentration of TAS occurring in the second week of life (*p* = 0.02; Table 4). The treatments did not differ in serum TBARS at birth, nor at the ninth week of life (*p* = 0.79, *p* = 0.49, respectively; Table 4), but there was an age effect, presenting the greatest values of TBARS at birth, and a decrease in concentration towards the end of the sampling period (*p* < 0.01; Table 4). No differences were observed for haptoglobin concentration between treatments at birth (*p* = 0.44) and at the ninth week of life (*p* = 0.88; Table 4). Nevertheless, there were differences in haptoglobin concentration related to the time of sampling, increasing the concentration of this acute phase protein towards the ninth week of life (*p* < 0.01).

Serum metabolites, glucose, total protein, and albumin did not present differences between calves born from cows from TG and CG (Table 4). However, total protein and albumin were different at different sampling times, increasing their concentrations towards the last sampling (*p* < 0.01). Despite this, no interaction was observed between treatments and time elapsed from birth to sampling, for any of the three metabolites analyzed (Table 4).

## 4. Discussion

While the phenomenon of accelerated fetal growth in late pregnancy is well established [42], our comprehension of the impact of maternal antioxidant status during this crucial period on fetal development, and subsequently, the postnatal implications for health and performance, is still evolving [43]. Substantial evidence suggests that maternal stress before parturition not only influences colostrogenesis, but also compromises fetal development, leading to postnatal repercussions, particularly in immunology, reproduction, and growth. These effects may persist until the first lactation in dairy cattle [13,44].

Despite existing research, to the best of our knowledge, our study marks a novel exploration, since it is the first to explore how supplementing the mother’s antioxidant system in the last third of gestation with injectable minerals and vitamins involved directly or indirectly in antioxidant defenses, impacts calf development during the first two month of life, in dairy systems.

Notably, all calves born from cows involved in the present study exhibited serum total protein concentrations surpassing 9.4° Brix within 48 h of colostrum intake, indicative of a successful transfer of passive immunity, aligning with the contemporary standards outlined by Lombard et al. [36]. Nevertheless, a noteworthy observation emerged as all calves, irrespective of their mothers’ treatment, experienced at least one episode of diarrhea during the second week of life. The vulnerability of calves during this period is accentuated by a decline in passive immune transfer and an immature immune system [45].

Despite calves in the CG exhibiting greater fecal scores throughout the experimental period, no effect of antioxidant treatment in the pregnant cows were observed on days to onset of the first episode, duration of the episodes of diarrhea, number of episodes of diarrhea, and intensity calculated as the number of days with a fecal score greater than three. The stress suffered at birth probably reduced the antioxidant capacity of the animal, and a reinforcement in the antioxidant pool in post-birth calves would have helped to improve these variables, as reported by Miqueo et al. [46]. On the other hand, it is probably that the lesser fecal score in TG calves plus the greater starter intake allowed them to reach the body weight of calves from the CG at the end of the trial, since CG calves have a tendency to present greater body weights at birth. Calves in TG could probably cope better with the metabolic challenges of the first period of life due to the effect of antioxidants on the immune system, but direct measurements were not conducted to be able to draw more accurate conclusions on this topic.

The greater fecal scores of calves in CG and the lesser starter intake compared to TG calves, did not impact body weight, average daily gain, growth measures, or feed efficiency. Other authors described an improvement in feed efficiency in feedlot calves when supplemented with trace minerals [47,48]. Also, an increase was not observed in rectal temperature, heart, or respiratory rates in CG animals. During the trial, those vital parameters consistently fell within the normal range as documented in the literature, exhibiting comparable trends to those observed by other researchers [49].

Mineral and vitamins are essentials in metabolic and growth process [50,51]. However, the mother’s parenteral treatment with trace minerals and vitamins seems to have not affected the growth performance of calves during the first two months of life. The bibliography presents conflicting findings on this matter. While Shao et al. [52] and Stokes et al. [32] at the finishing phase of steers, did not observe effects on the dam’s supplementation with trace minerals, Marques et al. [53] reported a treatment effect on weaning body weight, particularly for calves born from beef cows supplemented with microminerals during the prepartum period. Those authors argued that it could be due to a fetal programming effect on the immune function of cows supplemented with microminerals. The absence of a treatment effect on the calves in our study may be attributed to multifaceted factors associated with the health challenges faced by animals at this critical stage of life [27], but also it could be related to the tighter control of the nutrients ingested during the prepartum period in dairy cows in comparison with prepartum beef cows.

Reactive oxygen substances are intrinsic byproducts of the metabolic processes in living organisms, occurring periodically as a natural consequence of their functionality. However, instances of stress significantly elevate the production of these substances, surpassing the organism’s intrinsic antioxidant defense mechanisms. This phenomenon is commonly referred to as oxidative stress, as extensively discussed by Eitam et al. [54] and Enriquez et al. [55]. While existing evidence highlights the impact of oxidative stress on the health, reproduction, and production of cows during the transition period, scant attention has been devoted to comprehending the effects of free radicals generated both by the mother prepartum and by the calf at birth and in its early months of life on the growth parameters of dairy calves [16,17,56]. Contrary to expectations, values of TBARS, used as estimators of lipid peroxidation, remained unaffected by the treatment during the prepartum period when measured on postpartum cows and calves at birth. But as reported by other authors, TBARS concentration was greater at birth, decreasing in the subsequent two sampling times [8,56,57]. Examining antioxidant status in newborn calves, some researchers reported a greater vulnerability of offspring to oxidative stress due to an immature immune system, consistent with our findings where newborn calves presented smaller TAS concentrations at birth, increasing by the last sampling time [56]. A noteworthy observation was the lower TAS concentration expressed during the second week of life, coinciding with the decrease in immunity acquired through colostrum ingestion in the first hours of life and leading to episodes of diarrhea [58].

The reference range for haptoglobin concentration in healthy animals is 10–20 mg/mL. However, during induced or naturally occurring inflammations, haptoglobin concentrations can surpass 20 mg/dL [59,60]. Postpartum dairy cows from our study had barely greater concentrations of haptoglobin than the upper limit suggested by the bibliography and without differences between treatments, but calves at birth presented values of haptoglobin in the normal range. Other authors published that haptoglobin concentrations greater than 20 mg/dL are associated with mild infection and above 40 mg/dL could be associated with severe infection [61]. In the present study, it was expected that haptoglobin concentration increased during the second week of life in CG calves, coinciding with the peak of diarrhea as in the study of Erling [62] and Peetsalu et al. [63], but calves from both the CG and TG presented no differences between treatments and the greater haptoglobin levels at the final sampling time (63 days). For his part, Schroedl et al. [64] found no differences in haptoglobin concentration in calves, in samples taken at birth, one day of age, and at 10 days of age in agreement with the results obtained in the current study. Other authors reported a reduction in the haptoglobin concentration, with the lowest values towards weaning, relating this lesser concentration with lower stress levels [65,66,67]. We probably did not observe this reduction in haptoglobin concentration in our experiment since the last sampling was made a week after the calves were weaned, and the change in feeding, added to the greater consumption of concentrate in that week, could have been responsible for the elevation in the haptoglobin concentration [68].

Although the differences observed between groups (e.g., fecal score and starter intake) are small, these differences could have some health effects at the herd level. In that sense, even minor variations in fecal scores and starter intake might impact overall herd health by affecting nutrient absorption and growth rates, which could in turn, influence long-term productivity and management strategies.

## 5. Conclusions

In summary, despite expectations, antioxidant treatment in pregnant cows did not affect diarrhea-related parameters in calves. It is possible that the stress at birth reduced the antioxidant capacity of the calves, suggesting a potential benefit of post-birth antioxidant reinforcement. Even though maternal supplementation with trace minerals and vitamins did not appear to significantly impact the neonatal calf’s antioxidant system, nor growth performance in the first two months, it may have subtle effects on calf growth and feed efficiency. Extending the study beyond the initial two months of a calf’s life could provide insights into any delayed effects of maternal antioxidant supplementation on the health, growth, and performance of the offspring. Overall, our findings contribute to the ongoing discussion on the role of maternal antioxidant status in calf development and underscore the complex interplay of factors influencing early-life health outcomes in dairy systems. Further research is warranted to elucidate the mechanisms underlying these observations and to optimize strategies for enhancing calf health and performance.

## Figures and Tables

**Table 1:** Nutrient composition, in dry matter basis, of diets offered to prepartum cows and calves during the trial.

	Prepartum 1 ^1^	Prepartum 2 ^2^	Ruter^® 3^	Mamon ^4^
Dry matter, %	32.7	40.85	92.48	90.55
Crude protein	18.15	9.39	29.6	22.77
ADF	32.17	28.46	08.01	09.09
aNDF	52.68	39.18	17.33	19.53
aNDFmo	49.29	34.7	14.25	16.45
Crude fat, %DM	3.21	2.30	6.63	5.35
Ash	10.85	11.84	8.29	08.03
Starch	3.19	12.96	24.26	29.77
NDT	65.46	58.1	77.36	76.20
McalME/KgDM ^5^	2.497	2.24	3.227	3.083

^1^ Prepartum dairy cows’ diet from 60 to 30 days to parturition, based in a fescue and white clover pasture. ^2^ Prepartum dairy cows’ diet from 30 days before calving to calving; total mix ration diet based on whole plant corn silage and which includes, among other ingredients, mineral salts mixed with the diet. ^3^ Starter concentrate (Ruter^®^, ACA, Buenos Aires, Argentina), fed to calves from birth to 21 days of life. ^4^ Starter concentrate (Mamon, Cooperativa Agropecuaria de Tandil Ltd.a., Buenos Aires, Argentina), fed to calves from 21 days to weaning. ^5^ NRC 2001 Energy calculations.

**Table 2:** Characteristics of the diarrhea episodes of calves born from cows with (TG) or without (CG) supplementation with mineral and vitamins during the prepartum period. Average fecal score, days to the onset of the first episode, duration of the episodes of diarrhea, number of episodes of diarrhea, and intensity calculated as the number of days with a fecal score greater than three. Rectal temperature, heart rate, and respiratory rate, measured periodically during the milk-feeding period.

	Treatments ^1^		SEM	*p*-Values ^2^		
	TG	CG		T	A	T*A
Diarrhea						
Fecal score	1.76	1.81	0.01	0.01	<0.01	0.96
Onset of diarrhea, d	8.90	8.61	0.36	0.58		
Duration of diarrhea, d	11.1	9.6	1.15	0.13		
Number of episodes	1.61	1.70	0.19	0.25		
Intensity of diarrhea ^3^	6.75	6.28	1.29	0.98		
Rectal temperature, °C	37.3	37.4	0.25	0.38	<0.01	0.88
Heart rate, beat/min	118	118	0.71	0.97	<0.01	0.22
Respiratory rate ^4^	35.1	34.9	1.31	0.61	<0.01	0.27

^1^ Treatments: TG: treatment (parenteral supplementation of minerals and vitamins: 5 mL of each, subcutaneously), CG: control (parenteral supplementation of sterile saline solution); ^2^ *p*-values: T: treatments (TG and CG), A: age, T*A: interaction of treatment × age; SEM: standard error of the median; ^3^ Number of days with a fecal score greater than three; ^4^ Respiratory rate (movement/minute).

**Table 3:** Dry matter intake (DMI), body weight, average daily gain (ADG), feed efficiency (ADG/Total DMI), and skeletal growth for calves born from cows supplemented with minerals and vitamins during prepartum.

	Treatments ^1^		SEM	*p*-Value ^2^		
	TG	CG		T	A	T*A
Intake, g DM/d						
Whole milk	668	670	1.07	0.14	<0.01	0.47
Average starter intake	540	522	37.9	0.04	<0.01	0.48
Total DMI	1266	1233	28.6	0.06	<0.01	0.47
Body weight, kg						
At birth	37.8	39.5	1.75	0.08	<0.01	0.99
Ninth week	76.7	77.8	2.87	0.57		
ADG, g/d	614	607	22.6	0.76	<0.01	0.88
Feed efficiency, g/g ^3^	0.47	0.47	0.003	0.94	<0.01	0.06
Skeletal size, cm						
Hip width				<0.01	<0.01	0.99
First week	20.9	21.4	0.44	0.03		
Ninth week	26.3	26.5	0.24	0.48		
Wither height				0.94	<0.01	0.99
First week	71.4	71.7	0.99	0.65		
Ninth week	82.9	83.2	1.32	0.68		
Heart girth				0.11	<0.01	0.95
First week	81.4	82.1	0.59	0.33		
Ninth week	99.1	100	1.31	0.28		
Skeletal growth, cm/d ^4^						
Hip with	0.20	0.19	0.01	0.56		
Wither height	0.08	0.08	0.01	0.40		
Heart girth	0.30	0.29	0.02	0.72		

^1^ Treatments: TG: treatment (parenteral supplementation of minerals and vitamins: 5 mL of each, subcutaneously), CG: control (parenteral supplementation of sterile saline solution); SEM: standard error of the median; ^2^ *p*-values: T: treatment (TG and CG), A: age (week), T*A: interaction between treatment x age; ^3^ Feed efficiency: calculated as average daily gain (ADG)/Total DMI; ^4^ Skeletal growth: calculated as the difference between the skeletal size in the ninth week and the first week of life, divided by the number of days (63 days).

**Table 4:** Oxidative stress biomarkers and haptoglobin in postpartum Holstein dairy cows and calves, and glucose, total protein, and albumin for calves born from cows supplemented with minerals and vitamins during the prepartum period.

	Treatments ^1^		SEM	*p*-Value ^2^		
	TG	CG		T	A	T*A
Cows						
TAS, mM	4.32	4.23	0.37	0.86		
TBARS, μM	5.18	4.55	0.43	0.32		
Haptoglobin, mg/dL	21.4	22.0	0.33	0.23		
Calves						
TAS, mM						
Birth	3.71	4.56	0.72	0.11	0.02	0.17
Ninth week	5.03	4.36	0.81	0.13		
TBARS, μM						
Birth	19.9	19.7	1.43	0.79	<0.01	0.64
Ninth week	6.52	7.27	1.710	0.49		
Haptoglobin, mg/dL						
Birth	19.8	19.6	0.33	0.44	<0.01	0.67
Ninth week	22.3	22.4	0.91	0.88		
Glucose, mg/dL	77.4	82.4	6.725	0.270	0.179	0.861
Total protein, g/dL	4.22	4.32	0.175	0.445	<0.01	0.556
Albumin, g/dL	2.09	2.14	0.100	0.440	<0.01	0.108

^1^ Treatments: TG: treatment, CG: control; SEM: standard error of the median; ^2^ *p*-values: T: treatment, A: age, T*A: interaction between treatment × age.

## Data Availability

Data will be shared upon request to the authors.
